# Stent Crush Bailout for a Deformed Stent Nodule Caused by Entrapped Guidewire Extraction: A Case Report

**DOI:** 10.7759/cureus.82980

**Published:** 2025-04-25

**Authors:** Ken Saito, Shuichi Yoneda, Hideo Matama, Teruo Noguchi

**Affiliations:** 1 Cardiovascular Medicine, National Cerebral and Cardiovascular Center, Suita, JPN

**Keywords:** bifurcation pci, calcified lesion, pot, stent deformation, wire entrapment

## Abstract

We report the case of an 82-year-old man with end-stage renal failure and complex coronary artery disease who underwent percutaneous coronary intervention (PCI) for severe calcified stenosis in the left main trunk and proximal left anterior descending artery. Given the heavily calcified nature of the lesion, rotational atherectomy (Rotablator, Boston Scientific, Marlborough, MA) was performed. A provisional stenting strategy was employed with side-branch wire protection. Protected hydrophilic-coated guide wire (Runthrough NS Ultra Floppy; Terumo, Tokyo, Japan) entrapment likely occurred due to wrapping around stent struts during proximal optimization technique (POT) in a heavily calcified bifurcation, resulting in stent deformation upon attempted wire withdrawal. Intravascular ultrasound played a crucial role in confirming wire position, stent deformation, and post-fenestration stent apposition. Final angiography demonstrated TIMI (Thrombolysis in Myocardial Infarction) 3 flow with no side-branch compromise, and the patient remained asymptomatic post-procedure. This case highlights the importance of meticulous technique and intravascular imaging guidance in high-risk bifurcation PCI.

## Introduction

Guidewire entrapment is a rare but recognized complication during percutaneous coronary intervention (PCI), particularly in bifurcation lesions with heavy calcification and complex anatomy. While side-branch wire protection is an essential technique in bifurcation PCI, it can occasionally lead to wire wrapping and entrapment [1]. One serious consequence of wire entrapment is stent deformation, which can compromise vessel patency and lead to procedural failure.

This case is unique in that a stent nodule, a focal protrusion of distorted stent struts, was formed as a result of wire extraction, necessitating a bailout stent crush strategy. Intravascular ultrasound (IVUS) played a crucial role in identifying the exact wire trajectory and guiding the bailout intervention, underscoring the importance of intravascular imaging in managing such complex scenarios.

Previous studies have shown that IVUS guidance improves outcomes in left main and bifurcation PCI by facilitating optimal stent expansion and identifying complications early [2,3]. In elderly patients with extensive calcified disease, as in the present case, bifurcation stenting presents additional challenges due to fragile vessel walls, impaired healing, and higher rates of mechanical complications. Thus, meticulous lesion preparation and intravascular imaging are essential to mitigate procedural risks.

Herein, we report a rare case of stent nodule formation due to wire entrapment during complex left main bifurcation PCI, managed successfully using IVUS-guided recrossing and stent crushing.

## Case presentation

**Figure 1:**
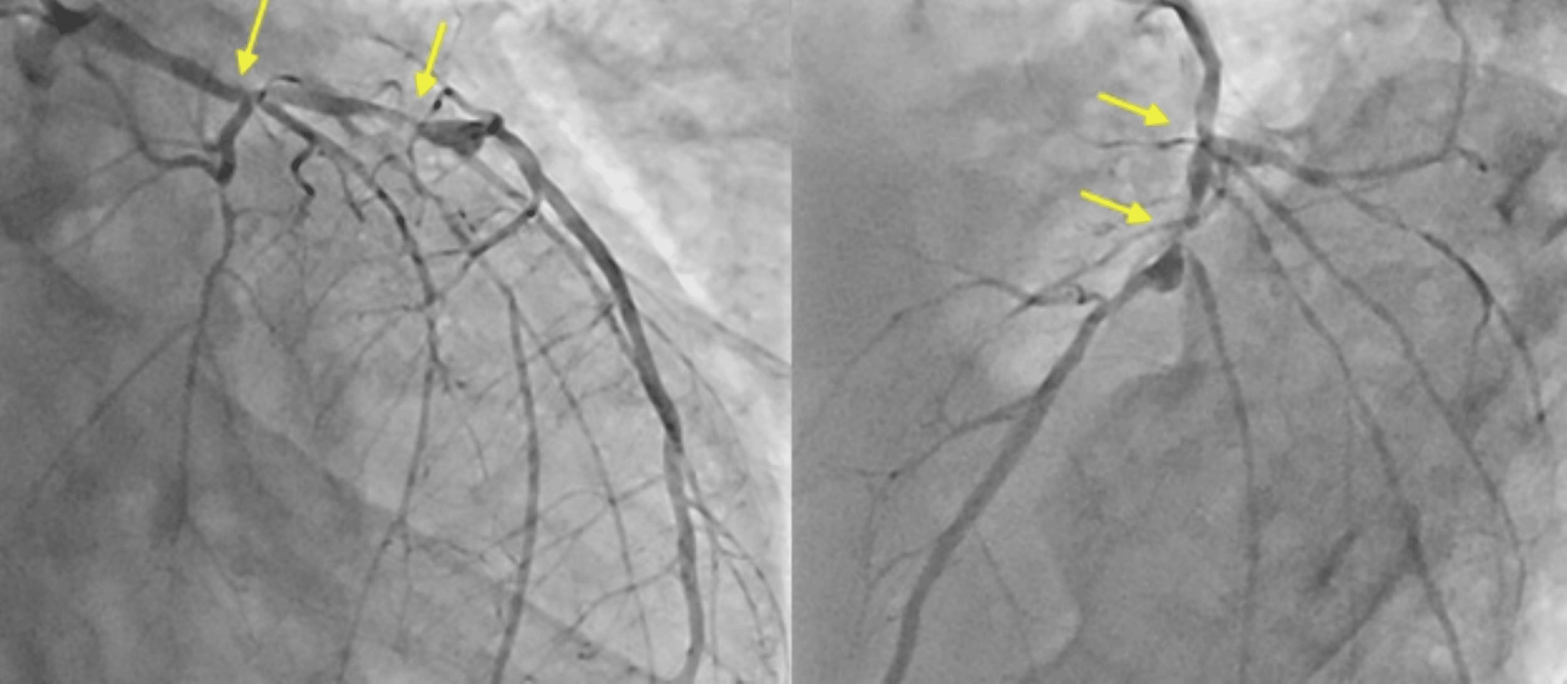
Baseline coronary angiography: Right anterior oblique caudal view and left anterior oblique cranial view

An 82-year-old man with end-stage renal failure, diabetes mellitus, hypertension, and dyslipidemia was admitted for acute heart failure. Coronary angiography revealed severe calcified stenosis in the distal left main trunk (LMT) extending into the proximal left anterior descending artery (LAD) (Figure 1).

Given the heavily calcified nature of the lesion, rotational atherectomy (Rotablator, Boston Scientific, Marlborough, MA) was performed using a 1.5 mm burr for adequate lesion preparation before stenting. After that, IVUS revealed 270° calcification at the LMT bifurcation, and the length of the LMT was approximately 35 mm.

A drug-eluting stent (DES) (Xience Skypoint 2.75/33 mm, Abbott Vascular, Chicago, IL) was deployed in the proximal LAD. A provisional stenting strategy was employed, with side-branch protection using guidewires: a Runthrough NS Ultra Floppy (UF; Terumo, Tokyo, Japan) for the left circumflex artery (LCX) and a Runthrough NS Izanai (Terumo, Tokyo, Japan) for the high lateral branch (HL). A second DES (Xience Skypoint 3.5/38 mm) was deployed from the LMT ostium to the proximal LAD to fully cover the long diseased segment. The stent diameter and length were selected to accommodate the vessel size and lesion length while minimizing the risk of edge restenosis.

**Figure 2:**
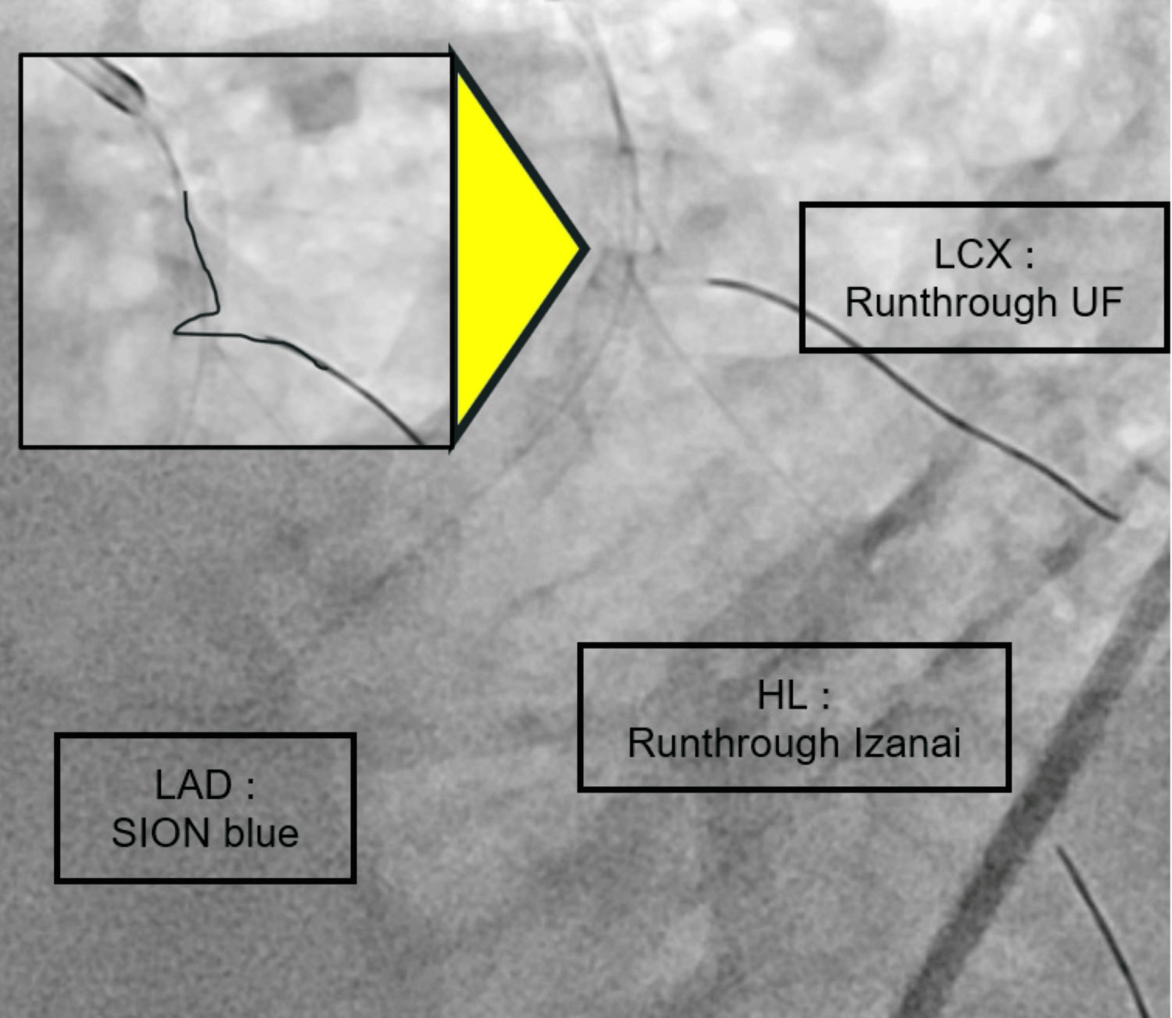
Entrapment of the protective guidewire (LAO cranial view) LCX: left circumflex artery; UF: Ultra Floppy; LAD: left anterior descending artery; HL: high lateral branch; LAO: left anterior oblique.

Following the proximal optimization technique (POT), resistance was encountered during the removal of the protective LCX wire. The wire appeared to be entrapped due to wrapping around the stent struts in a helical fashion, possibly as a result of torque buildup in the calcified bifurcation segment (Figure 2).

**Figure 3:**
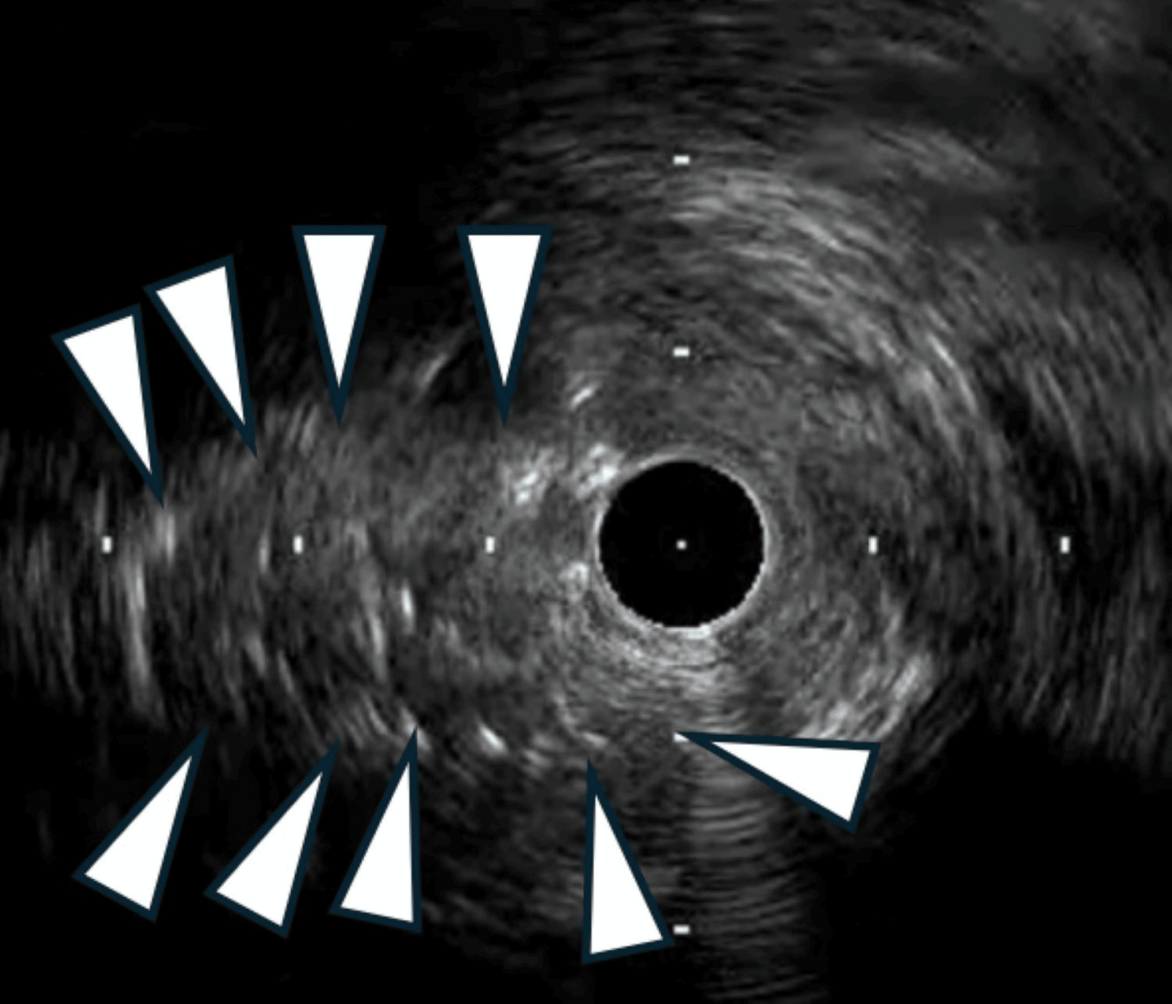
Intravascular ultrasound confirming the guidewire advanced outside the stent nodule (white arrowhead)

Attempts to pass a 1.0 mm balloon outside the DES failed, and the jailed wire was retrieved using the microcatheter-assisted technique (Caravel; Asahi Intecc, Aichi, Japan). However, the stent in the LMT was severely deformed, with the distal portion stretched proximally and a nodule formed at the ostium. During this maneuver, the main LAD wire was unintentionally dislodged, likely due to tension generated during manipulation of the jailed wire and catheter system. A new Gladius EX (Asahi Intecc, Aichi, Japan) polymer-jacketed guidewire was advanced outside the stent nodule and successfully recrossed into the distal lumen. IVUS revealed significant stent distortion at the LMT ostium, as well as the outside position of the wire relative to the stent structure (Figure 3).

**Figure 4:**
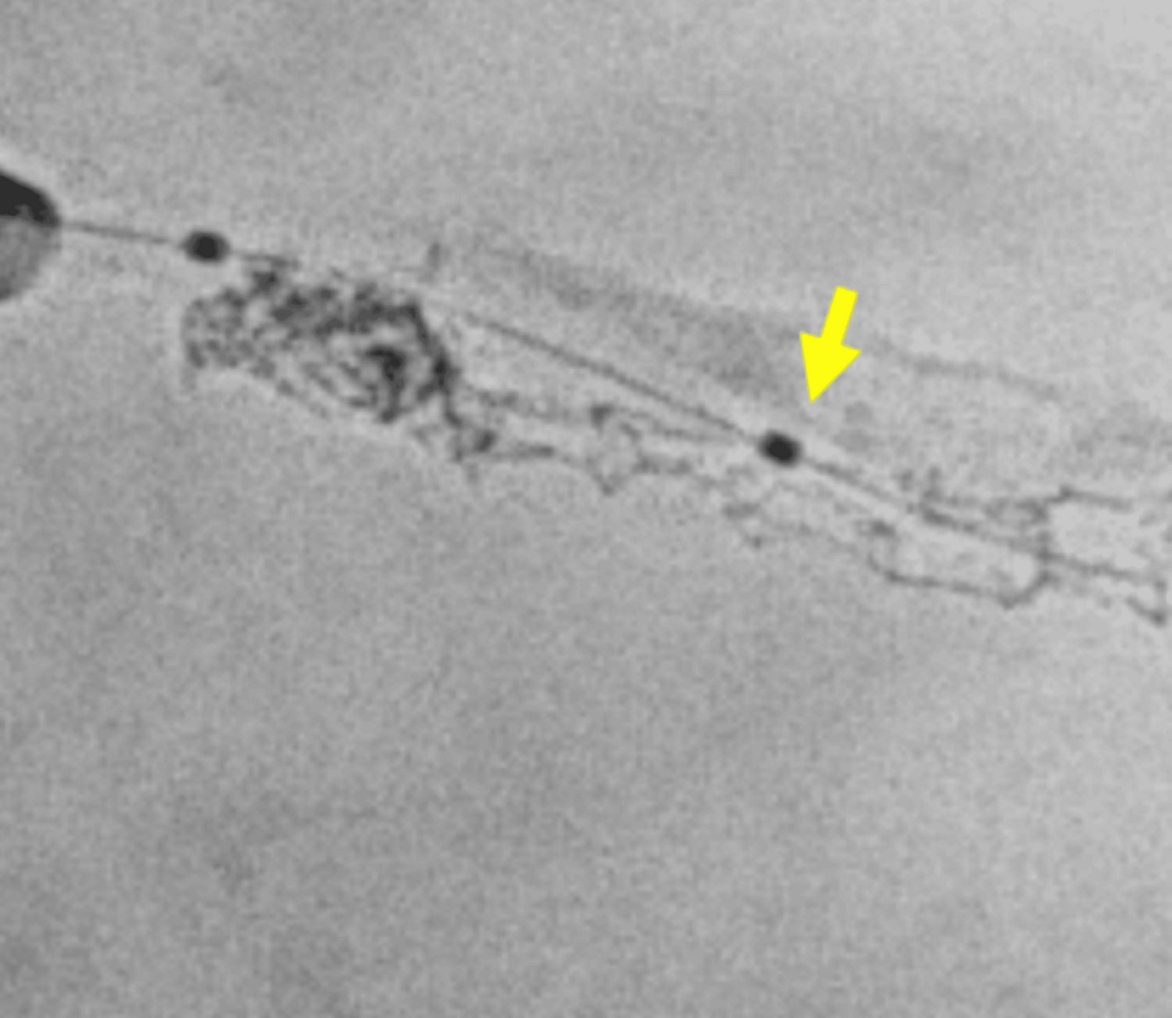
Balloon dilation performed to compress the stent nodule and fenestrate at the recross point (yellow arrow)

Balloon dilation was performed to compress the stent nodule against the vessel wall and fenestrate it at the recross point (Figure 4).

**Figure 5:**
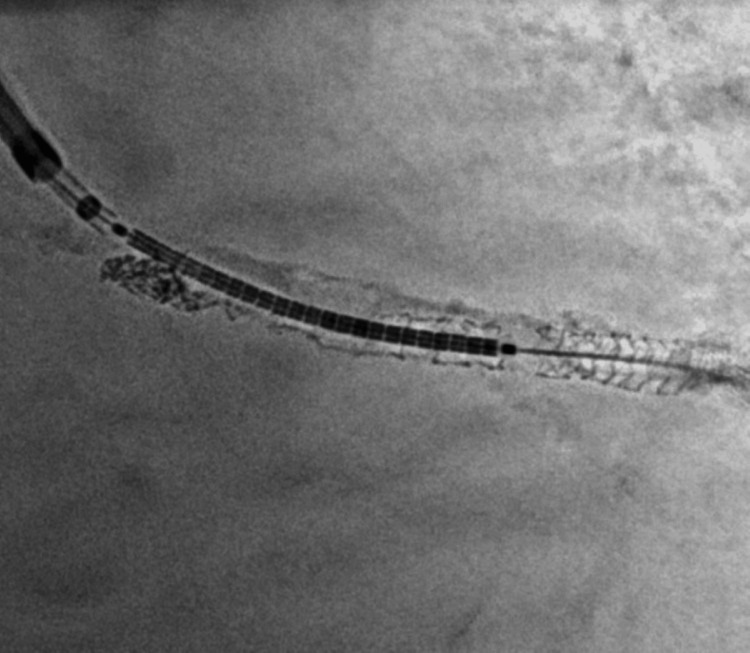
Deployment of a new drug-eluting stent

A new DES (Synergy Megatron 3.5/28 mm; Boston Scientific, Marlborough, MA) was placed from the LMT ostium to the proximal LAD (Figure 5).

**Figure 6:**
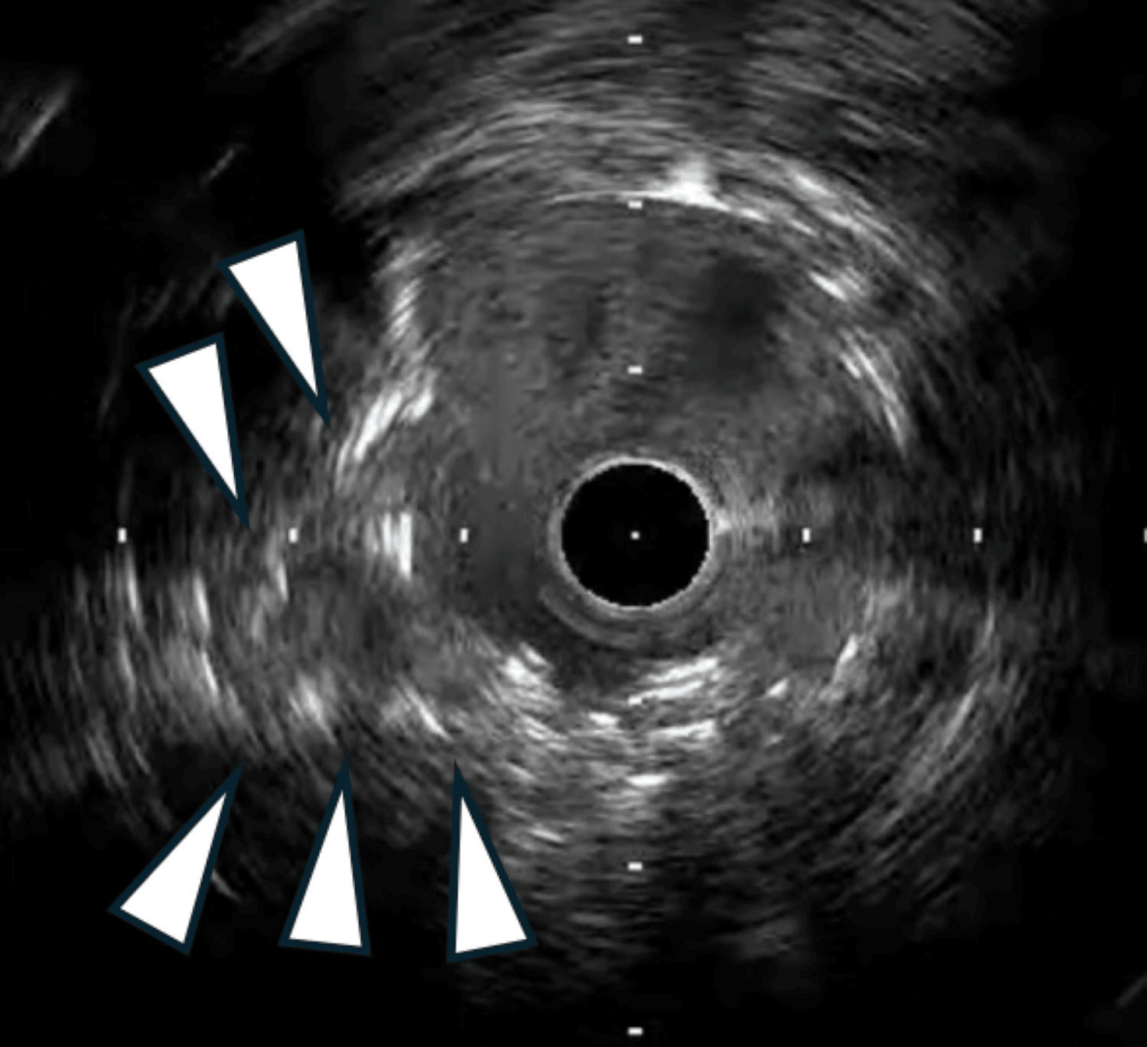
Intravascular ultrasound confirmed compressing stent nodule (white arrowhead)

Subsequently, IVUS confirmed that the newly deployed DES had successfully compressed the stent nodule, and the minimum stent area was 6 mm² (Figure 6).

**Figure 7:**
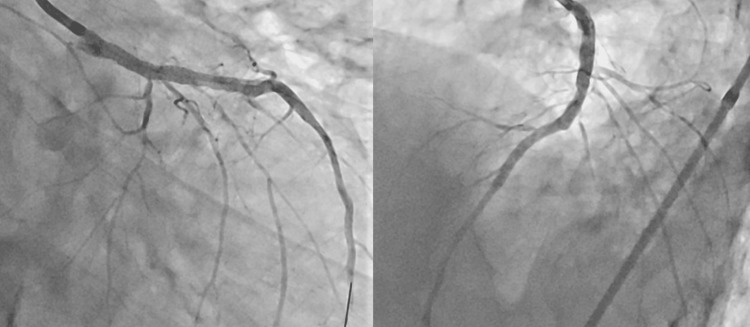
Final CAG (RAO caudal view and LAO cranial view) CAG: coronary angiography; RAO: right anterior oblique; LAO: left anterior oblique.

Final angiography confirmed good flow and no side-branch occlusion (Figure 7).

The patient remained hemodynamically stable throughout the procedure. No chest pain or ECG changes were noted post-PCI, and cardiac biomarkers remained within normal limits. Dual antiplatelet therapy (aspirin 100 mg and prasugrel 3.75 mg daily) was initiated. The patient was monitored for five days and discharged without complications. At one-month follow-up, he remained asymptomatic with no evidence of restenosis on repeat non-invasive imaging.

## Discussion

Wire entrapment during bifurcation PCI is rare but potentially serious, particularly in the presence of severe calcification and tortuous anatomy. In our case, entrapment was likely due to a combination of wire looping within the stent struts and increased friction during removal, exacerbated by vessel calcification. Although not clearly visible angiographically, the helical pattern of the LCX wire was suspected based on procedural resistance and the mechanical behavior observed during withdrawal. 

POT has become standard in bifurcation PCI to enhance stent expansion and facilitate re-crossing. However, in calcified vessels, aggressive ballooning can inadvertently trap the side-branch wire. In our case, a non-compliant balloon (4.0/12 mm) was inflated at 18 atm within the distal LMT. The resultant strut expansion and vessel wall interaction may have increased frictional resistance around the side-branch wire, predisposing it to entrapment. A more conservative POT using a slightly shorter balloon or reduced inflation pressure might have reduced this risk.

Multiple bailout strategies have been reported for wire entrapment, including microcatheter-assisted retrieval, rotational atherectomy, snare techniques, and even surgical removal [4,5]. We opted for microcatheter-assisted retrieval using the Caravel due to its lower risk of vessel injury compared to rotational atherectomy or snaring, particularly in a heavily calcified LMT. This technique also preserved control over the wire system and minimized procedural delay.

Unintentional withdrawal of the LAD wire during manipulation was a significant technical complication. It likely occurred due to the traction generated during the jailed wire retrieval process. This event emphasizes the importance of securing the main vessel wire.

To our knowledge, stent crushing to treat a wire-induced stent nodule in the LMT is extremely rare. Only a few similar cases involving stent deformation during wire retrieval have been documented [6]. Our approach of re-crossing outside the deformed stent struts and re-stenting appears to be a novel bailout method in this anatomical context. Although alternatives such as high-pressure ballooning or snaring were considered, we favored re-stenting for its reliability and ability to fully appose the stent nodule to the vessel wall.

IVUS was indispensable throughout the procedure. It helped confirm that the re-crossed wire passed outside the stent nodule, identified the exact location and extent of stent deformation, and verified stent apposition after bailout stenting. 

This case highlights several practical considerations for bifurcation PCI in calcified vessels: careful shape of side-branch wires, cautious use of POT in long calcified lesions, and the importance of intravascular imaging for procedural guidance and complication management. 

## Conclusions

This case highlights a rare but serious complication of bifurcation PCI involving wire entrapment and stent deformation in a calcified LMT lesion. The use of IVUS was instrumental in guiding wire re-crossing and confirming stent expansion after the bailout intervention. The complication was successfully managed using a stent crush technique, in which a new stent was deployed to compress and fenestrate the deformed segment caused by wire retrieval.

Several key learning points emerge from this case. First, the POT, while beneficial for stent apposition, may increase the risk of wire entrapment in long, heavily calcified bifurcation segments if not performed with caution. Second, unintentional wire loss, particularly of the main vessel wire, can significantly complicate bailout procedures and should be proactively prevented through wire anchoring and maintaining sufficient slack. Third, IVUS provides essential real-time insights that facilitate decision-making in complex bailout situations.

To reduce the risk of wire entrapment, operators should consider careful POT technique with appropriate balloon sizing and length, and avoid excessive pressure. This experience reinforces the need for meticulous planning, vigilant wire handling, and liberal use of imaging guidance in high-risk LMT bifurcation PCI.

Given the scarcity of reported cases, documentation of similar complications in procedural registries may help refine best practices and stimulate future research into preventive strategies and device optimization.
